# Transcriptional Repression of the *Dspp* Gene Leads to Dentinogenesis Imperfecta Phenotype in *Col1a1-Trps1* Transgenic Mice

**DOI:** 10.1002/jbmr.1636

**Published:** 2012-04-16

**Authors:** Dobrawa Napierala, Yao Sun, Izabela Maciejewska, Terry K Bertin, Brian Dawson, Rena D'Souza, Chunlin Qin, Brendan Lee

**Affiliations:** 1 Institute of Oral Health Research, Department of Oral and Maxillofacial Surgery, School of Dentistry, University of Alabama at BirminghamBirmingham, AL, USA; 2 Department of Molecular and Human Genetics, Baylor College of MedicineHouston, TX, USA; 3 Department of Biomedical Sciences, Baylor College of Dentistry, Texas A&M University Health Science CenterDallas, TX, USA; 4 Howard Hughes Medical Institute, Baylor College of MedicineHouston, TX, USA

**Keywords:** MINERALIZATION, DENTINOGENESIS, TRPS1, DSPP, TRANSCRIPTION

## Abstract

Dentinogenesis imperfecta (DGI) is a hereditary defect of dentin, a calcified tissue that is the most abundant component of teeth. Most commonly, DGI is manifested as a part of osteogenesis imperfecta (OI) or the phenotype is restricted to dental findings only. In the latter case, DGI is caused by mutations in the *DSPP* gene, which codes for dentin sialoprotein (DSP) and dentin phosphoprotein (DPP). Although these two proteins together constitute the majority of noncollagenous proteins of the dentin, little is known about their transcriptional regulation. Here we demonstrate that mice overexpressing the Trps1 transcription factor (*Col1a1-Trps1* mice) in dentin-producing cells, odontoblasts, present with severe defects of dentin formation that resemble DGI. Combined micro–computed tomography (µCT) and histological analyses revealed tooth fragility due to severe hypomineralization of dentin and a diminished dentin layer with irregular mineralization in *Col1a1-Trps1* mice. Biochemical analyses of noncollagenous dentin matrix proteins demonstrated decreased levels of both DSP and DPP proteins in *Col1a1-Trps1* mice. On the molecular level, we demonstrated that sustained high levels of Trps1 in odontoblasts lead to dramatic decrease of *Dspp* expression as a result of direct inhibition of the *Dspp* promoter by Trps1. During tooth development *Trps1* is highly expressed in preodontoblasts, but in mature odontoblasts secreting matrix its expression significantly decreases, which suggests a Trps1 role in odontoblast development. In these studies we identified Trps1 as a potent inhibitor of *Dspp* expression and the subsequent mineralization of dentin. Thus, we provide novel insights into mechanisms of transcriptional dysregulation that leads to DGI. © 2012 American Society for Bone and Mineral Research.

## Introduction

Dentin, one of the four major components of teeth, is a calcified tissue produced by neural crest–derived cells, odontoblasts, that form a continuous single-cell layer at the periphery of the dental pulp.^1^ The functional odontoblast has a columnar shape with a long cellular process and its major function is to secrete matrix and regulate matrix mineralization. Polarization of odontoblasts is required for the site-specific secretion of different components of extracellular matrix, and is a key factor in specifying the site of extracellular matrix mineralization.^2^ Newly synthesized, unmineralized matrix is referred to as predentin. Predentin separates the odontoblast layer from the mineralized matrix, dentin. Dentin is similar to bone in its matrix protein composition^3^; however, unlike bone, it does not undergo remodeling and does not participate in calcium homeostasis. The molecular hallmark of dentin is a high abundance of dentin sialophosphoprotein (DSPP), which is essential for dentin development and mineralization.

The most common hereditary defect of dentin is dentinogenesis imperfecta (DGI), which is a genetically and clinically heterogeneous disorder. Type I DGI (DGI-I) is associated with forms of osteogenesis imperfecta (OI) caused by mutations in one of the type I procollagen genes, *COL1A1* or *COL1A2*, whereas the more severe DGI-II and DGI-III are isolated dentin disorders caused by mutations in the *DSPP* gene.^4^, ^5^ Whereas mutations in these genes coding for the most abundant dentin proteins are responsible for the majority of DGI cases, the etiology of other genetic disorders manifesting dentin abnormalities is still unknown.^6^, ^7^

The *DSPP* gene encodes dentin sialophosphoprotein, a member of the Small Integrin-Binding Ligand, N-Glycosylated (SIBLING) family of extracellular matrix proteins. The SIBLING family consists of dentin matrix protein 1 (DMP1), dentin sialophosphoprotein (DSPP), bone sialoprotein (BSP), osteopontin (OPN), and matrix extracellular phosphoglycoprotein (MEPE).^8^ SIBLING proteins undergo extensive posttranslational modifications that include phosphorylation and glycosylation.^9^ In addition to these modifications, the DSPP peptide is proteolytically cleaved to form dentin sialoprotein (DSP) and dentin phosphoprotein (DPP).^10^ DSP and DPP are the most abundant noncollagenous dentin proteins that play distinct roles in dentin mineralization. In vivo mouse model studies have demonstrated that DSP is involved in the initiation of dentin deposition and mineralization, whereas DPP is critical for mineral maturation.^11^ Thus far, among the genes expressing the noncollagenous dentin proteins, only mutations in the *DSPP* gene have been found in inherited dentin disorders.

Tooth defects similar to the DGI-III phenotype have been demonstrated in *Dspp* null mice.^12^ The dental phenotype of *Dspp* null mice is characterized by: hypomineralization, an enlarged pulp cavity, a widened predentin layer, and a reduced zone of dentin. Defective tooth mineralization and dentin formation were also observed in a number of knockout and odontoblast-specific transgenic mice. In a majority of these mice, decreased *Dspp* levels were observed as well, underscoring the importance of transcriptional regulation, posttranslational processing, and signal transduction of DSPP for dentin formation. The expression of many signaling molecules has been detected during odontoblast differentiation and their effect on the expression of dentin-specific genes has been studied. Transforming growth factor β (TGF-β), bone morphogenic protein (BMP), fibroblast growth factor (FGF), and Wnt signaling have been demonstrated to affect the expression of *Dspp* and odontoblast differentiation.^13^–^17^ However, the transcription factors that mediate responses to these signals during dentinogenesis in vivo are unknown. Promoters of the *Dspp* and *Dmp1* genes and elements driving tissue-specific expression of these genes have been well characterized. Several transcription factors, including those with well-established roles in tooth development, have been demonstrated to interact with these promoters in vitro.^18^–^20^ Interestingly, the *Dspp* promoter, but not the *Dmp1* promoter, contains an abundance of potential binding sites for GATA transcription factors.^19^, ^20^

TRPS1 is a GATA-type transcription factor expressed during tooth development.^21^ In humans, mutations in the *TRPS1* gene cause an autosomal dominant craniofacial and skeletal dysplasia: tricho-rhino-phalangeal syndrome (TRPS).^22^ TRPS patients have characteristic facies, sparse hair, and skeletal abnormalities characteristic of disturbed endochondral bone formation. In addition, TRPS patients can present with dental phenotypes that include microdontia, malocclusion, delayed tooth eruption, and supernumerary teeth.^21^ During early tooth development, *Trps1* is highly expressed in dental mesenchyme and after cell differentiation, in dental papilla and preodontoblasts. Interestingly, *Trps1* expression is significantly decreased in differentiated secretory odontoblasts, which suggests a role for *Trps1* in odontoblast maturation.^21^ However, its role in postnatal odontoblast development and function cannot be studied using a conventional gene disruption strategy, because *Trps1* homozygous mutant mice die at birth.^23^, ^24^ Previous analyses of skeletal abnormalities in *Trps1* mutant mice revealed that *Trps*1 is involved in chondrocyte proliferation and differentiation.^24^–^26^ We and others have demonstrated that Trps1 acts as a transcriptional repressor that antagonizes Ihh signaling during endochondral bone formation.^25^, ^26^ Moreover, we have shown that the processes of chondrocyte development and mineralization of perichondrium were uncoupled in *Trps1*^*ΔGT/ΔGT*^ mice, resulting in advanced mineralization of perichondrium. This phenotype was corrected in *Trps1*^*ΔGT/+*^*;Runx2*^*+/−*^ double heterozygous mice demonstrating the genetic interaction between the Trps1 and Runx2 transcription factors in developing endochondral bones.^25^

In these studies, using a transgenic mouse approach, we address the role of *Trps1* in mineralization. We demonstrate that sustained high expression of *Trps1* in mature odontoblasts has a deleterious effect on their function. Using a combination of histological, biochemical, and molecular analyses we discovered that Trps1 directly targets the *Dspp* gene and represses its expression in vivo, leading to a DGI-like phenotype in transgenic mice.

## Materials and Methods

### Generation of *Col1a1-Trps1* transgenic mice and maintenance of mouse colony

We cloned the full-length V5-tagged Trps1 cDNA under the 2.3-kb fragment of the collagen type I promoter in a vector containing a tyrosinase expression cassette.^27^ Mice were maintained on an FVB/N background and housed in the Baylor College of Medicine Transgenic Mouse Facility according to standards of the Center of Comparative Medicine in full compliance with all applicable federal and state guidelines. Transgenic mice were identified by eye and fur color, and the genotypes were confirmed in the offspring of founder mice by PCR with primers specific for the *Trps1* cDNA (gcagcatatgccagcatctttg) and WPRE sequence (actgacaattccgtggtgttgtcg). Transgenic mice were fed with a soft diet produced by milling of the standard mouse diet (Teklad, Harlan Laboratories Inc., Indianapolis, IN, USA). All studies were performed with prior approval of Institutional Animal Care and Use Committee.

### Micro–computed tomography analyses

We fixed mouse heads in formalin for 48 hours followed by 70% ethanol washes and scanned using ScanCo micro–computed tomography (µCT)-40 instrument with 16-µm resolution. Images were reconstructed and analyzed using ScanCo software. All cross-section images of incisors were taken from the region located 0.8 mm posterior to the anterior terminus of the bone dorsal to the incisor.

### Histology, immunohistochemistry, and RNA in situ hybridization

We fixed mouse heads in 4% paraformaldehyde. For P7 mice and older, heads were decalcified in 10% EDTA prior to paraffin embedding; 7-µm sections were stained with hematoxylin and eosin (H&E) according to standard protocols. Undecalcified tissues were embedded in methyl methacrylate; 5-µm sections were stained with either von Kossa reagent or Alizarin Red according to standard protocols. Immunohistochemistry (IHC) was done using enzymatic antigen retrieval and antibodies: anti-Trps1 (Abnova, Taipei, Taiwan, 1:50 dilution), anti-osteocalcin (Abcam, Cambridge, UK, 1:200 dilution), and anti-TNAP (Abcam, 1:100 dilution); and aminoethyl carbazole (AEC) chromogen detection (Invitrogen, Carlsbad, CA, USA). In situ hybridization (ISH) was performed as described.^28^ The generation of the *Trps1* probe was as described.^25^ The *Dspp* probe was generated as described for the rat *Dspp*.^29^ The *Dmp1* probe was generated by PCR amplification with primers specific for *Dmp1* cDNA (F: aagactgtcattctccttgtg, R: cccgtactcttagagagtcc) and subsequent cloning of the PCR product into the EcoRV site of the pBluescriptK/S(+) plasmid. The antisense and sense *Dmp1* probes were generated by in vitro transcription with T3 and T7 polymerases, respectively.

### Analyses of SIBLING proteins

We performed extraction and analyses of dentin matrix proteins as described in detail.^30^, ^31^ Briefly, proteins were extracted from the dentin of 3-month-old males (*n* = 8 each group). For each analyzed genotype total protein extracts were combined and equal amounts of proteins from the wild-type (WT) and transgenic dentin were used in the comparative analyses. After removal of collagen fraction, noncollagenous proteins were separated into a total of 120 sequential elution fractions by a Q-sepharose ion-exchange column connected to a fast liquid protein chromatography (FLPC) system. Fractions 31 to 55 containing different types of the SIBLING proteins were analyzed on an SDS-PAGE gel and stained with Stains-All to visualize the proteins. This standardized procedure allows for comparison of the relative levels of SIBLING proteins in the analyzed samples.^30^–^32^ DSP protein was detected by Western blot with monoclonal anti-DSP 2G7.3 antibodies.^33^

### Generation of stable cell lines using piggyBac transposon system

PiggyBac-expressing vector pCMV-*piggyBac* and transposon constructs were kindly provided by Dr. Mathew Wilson^34^ (Baylor College of Medicine). MDPC-23 cells were transfected with 1:5 ratio of transposase-expressing:transposon plasmids using Lipofectamine 2000 according to the manufacturer's protocol. Puromycin selection (5 µg/mL) of transposon-positive cells was begun 48 hours after transfection.

### RNA extraction, cDNA synthesis, and qPCR for gene expression and chromatin immunoprecipitation

We extracted RNA using Trizol according to the manufacturer's protocol. One microgram (1 µg) of total RNA after DNase I treatment was converted to cDNA with a SuperScript III Reverse Transcriptase kit (Invitrogen). Quantitative PCR assays were performed using a LightCycler and FastStart DNA Master SYBR Green reaction mix (Roche, Indianapolis, IN, USA).

### Chromatin immunoprecipitation assay

We performed chromatin immunoprecipitation (ChIP) using a Chromatin Immunoprecipitation Assay Kit (Millipore, Billerica, MA, USA) according to the manufacturer's protocol. V5 antibodies (Invitrogen) were used to immunoprecipitate V5-tagged Trps1-DNA complexes and normal mouse immunoglobulin G (IgG) (Santa Cruz, Santa Cruz, CA, USA) was used as a specificity control. Primers for ChIP analyses were designed to detect immunoprecipitated chromatin containing either one potential GATA binding site (fragments A and B) or two sites (fragment C). *GAPDH* was used as a specificity control. Primer sequences are available upon request. The ChIP assay was performed in triplicate.

### Luciferase reporter Assay

We generated luciferase reporter constructs by PCR amplification of the mouse *Dspp* promoter fragments with the following forward primers F1: caggtagaactccatgagtttcag, F2: ctatggaagtgcagcttcgagg, F3: gttcaggcaagtactttttcc, F4: tacctcaggaatgataggggtc; and a reverse (R) primer: tgagagtggcacactgtcctg. PCR products were cloned into the SmaI restriction site of pGL3 basic vector (Promega). Point mutations in selected putative GATA consensus elements were generated by site-specific mutagenesis (QuikChange II Site-Directed Mutagenesis Kit; Stratagene, La Jolla, CA, USA) using the following mutagenesis oligonucleotides: M1 tgtacctcaggaataataggggtcttaaatagcc, M2 gatatatcacactgattatatatatttaagacacaaaa, M3 ctttaaacccccacataagggatcctaagcagtgattg, M4 cagggatcctaagcagttattggttgagaaaattatc. Reporter constructs were cotransfected with a plasmid expressing renilla luciferase (phRL-null; Promega, Madison, WI, USA). Luciferase activity was assayed 48 hours after transfection.

### Statistical analysis

Statistical analyses of the weight of the 3-week-old and 4-week-old mice were done using one-way ANOVA and comparison to control (WT mice) was made using Dunnett's test. For the 5-week-old and 6-week-old mice, ANOVA on ranks was performed because the data were not normally distributed, and the Dunnett's test was used to compare the various groups to the control (WT mice). All other statistical analyses were done using paired *t* test.

## Results

### Generation and characterization of *Col1a1-Trps1* transgenic mice

**Fig. 1:**
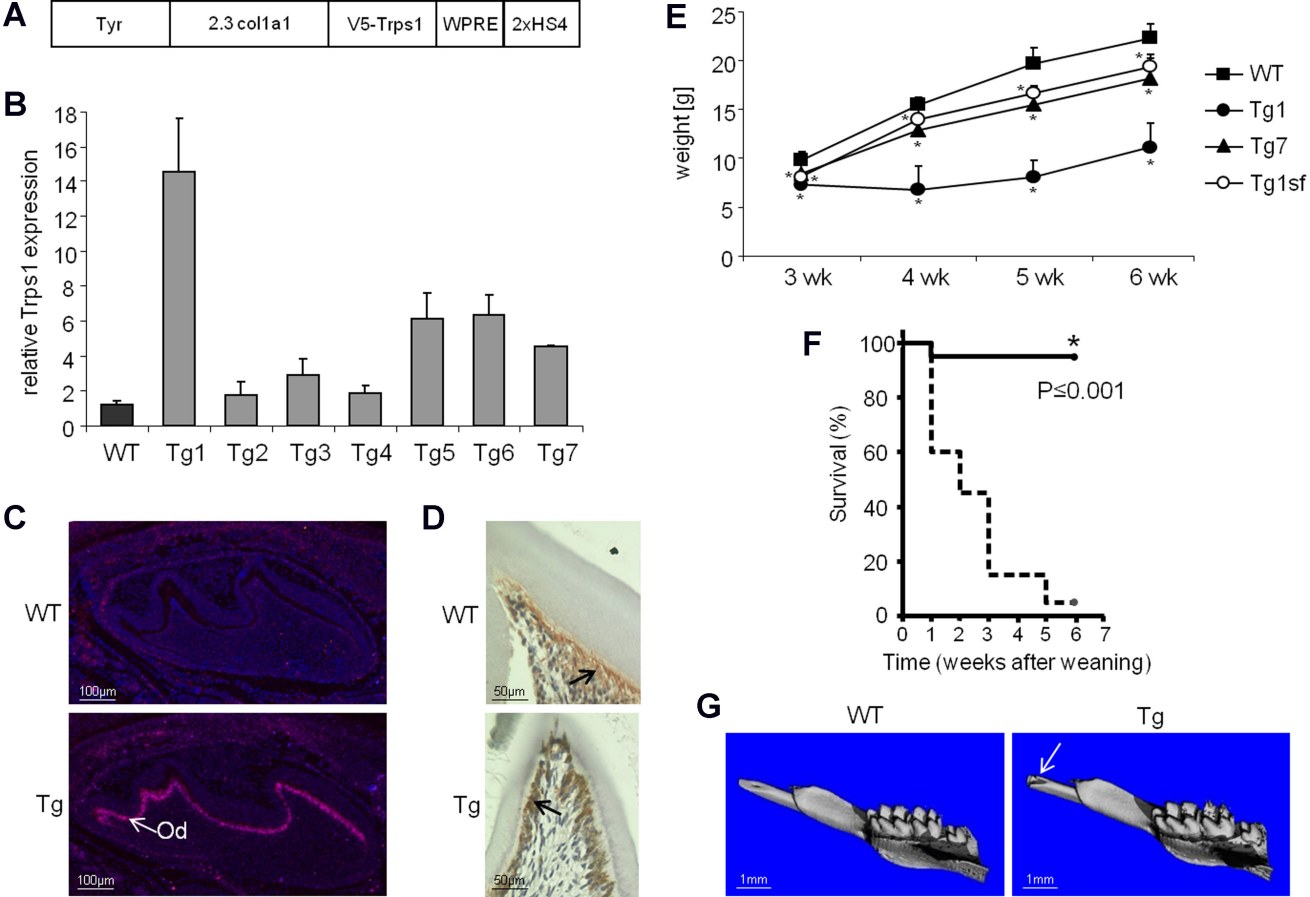
Gross characterization of *Col1a1-Trps1* transgenic mice. (*A*) Schematic of the transgenic construct: Tyr = tyrosinase cassette containing tyrosinase promoter and cDNA for coat/eye color identification of transgenic animals^27^; 2.3 col1a1 = 2.3 kb fragment of the *Col1a1* promoter; V5-Trps1 = V5-tagged *Trps1* cDNA; WPRE = woodchuck hepatitis virus posttranscriptional regulatory element; 2xHS4 = 2 copies of HS4 chromatin insulator. (*B*) Analyses of *Trps1* expression in calvarium of WT and *Col1a1-Trps1* mice by qRT-PCR (P2). Bars represent mean value of relative *Trps1* expression in WT mice and seven transgenic lines (Tg) normalized to GAPDH expression (*n* = 3). The value of *Trps1* expression in WT animals was arbitrarily set to 1. Error bars represent standard deviations. (*C*) Analyses of *Trps1* expression in teeth of WT and *Col1a1-Trps1* mice (P2) by RNA in situ hybridization. Arrows indicate *Trps1* expression in transgenic odontoblasts (Od). (*D*) Analyses of Trps1 expression in teeth of WT and *Col1a1-Trps1* mice (P14) by immunohistochemistry. Arrows indicate Trps1 in odontoblasts. (*E*) Weight curves of WT and *Col1a1-Trps1* mice (males) demonstrate growth retardation of transgenic animals. The phenotype is more severe in the transgenic line Tg1 that overexpresses Trps1 at higher levels than the Tg7 line. The growth retardation is corrected by providing soft food diet (open circles: Tg1 transgenic males fed with soft food). Data are shown as mean (*n* = 10 for each group except Tg1 4wk [4 weeks old], *n* = 6; Tg1 5wk [5 weeks old] and Tg1 6wk [6 weeks old], *n* = 4), error bars represent standard deviation, *p ≤ 0.05. (*F*) Kaplan-Mayer survival curve demonstrating postweaning lethality of *Col1a1-Trps1* mice (*n* = 20 each group). The experiment was terminated at 6 weeks after weaning. (*G*) µCT images showing broken incisors (arrow) in *Col1a1-Trps1* mice (3 weeks old).

In our previous studies on the role of the Trps1 transcriptional repressor during endochondral bone formation, we demonstrated that *Trps1* deficiency results in advanced mineralization of perichondrium. These data suggest that Trps1 acts as a negative regulator of mineralization. To address the role of the Trps1 transcription factor in the mineralization process in vivo, we generated transgenic mice expressing V5-tagged *Trps1* from a cell-type specific *Col1a1* promoter (*Col1a1-Trps1* mice) that specifically drives transgene expression in osteoblasts and odontoblasts.^35^ To simplify phenotypic discrimination of transgenic animals by fur and eye color we included a marker gene–expressing cassette in our transgenic construct (Fig. 1*A*). This additional cassette contains the tyrosinase cDNA expressed from its endogenous promoter independently of the *Col1a1-Trps1* expression.^27^ The tyrosinase minigene is also expressed in odontoblasts, resulting in pigment deposition in these cells; however, it does not impair dentin formation (Supplemental Fig. S1).

We first analyzed all transgenic lines for levels of *Trps1* expression in calvaria by quantitative RT-PCR on mRNA isolated from P2 mice (Fig. 1*B*). For detailed analyses of dental and skeletal phenotype we selected two lines (with different overexpression levels of *Trps1*) from a total of seven transgenic lines generated. The Tg1 line is the higher overexpressing line with *Trps1* mRNA levels in calvaria 15 times higher than in WT littermates. As a second line we chose line Tg7 with lower levels of the *Trps1* overexpression (4.5 times more than WT littermates). The phenotype of these transgenic lines is similar and the severity of abnormalities is directly correlated with the level of transgene overexpression. *Col1a1-Trps1* mice are normal at birth and show no apparent defects during the first 3 weeks of life. However, shortly after weaning they present with a severe growth retardation phenotype and demonstrate a high incidence of lethality (Fig. 1*E*, *F*; Supplemental Fig. S2).

Additionally, for the Tg1 line we confirmed the transgene overexpression in odontoblasts using RNA ISH and IHC (Fig. 1*C*, *D*). Analyses of *Trps1* expression at the protein level revealed that Trps1 persists at low levels throughout the life of odontoblasts in WT mice. Thus, in WT mice *Trps1* is highly expressed in developing odontoblasts, whereas in secretory odontoblasts its expression is maintained at low levels. In contrast, in transgenic mice high expression of *Trps1* is sustained in functional odontoblasts.

### Overexpression of *Trps1* in odontoblasts causes defective dentin formation

**Fig. 2:**
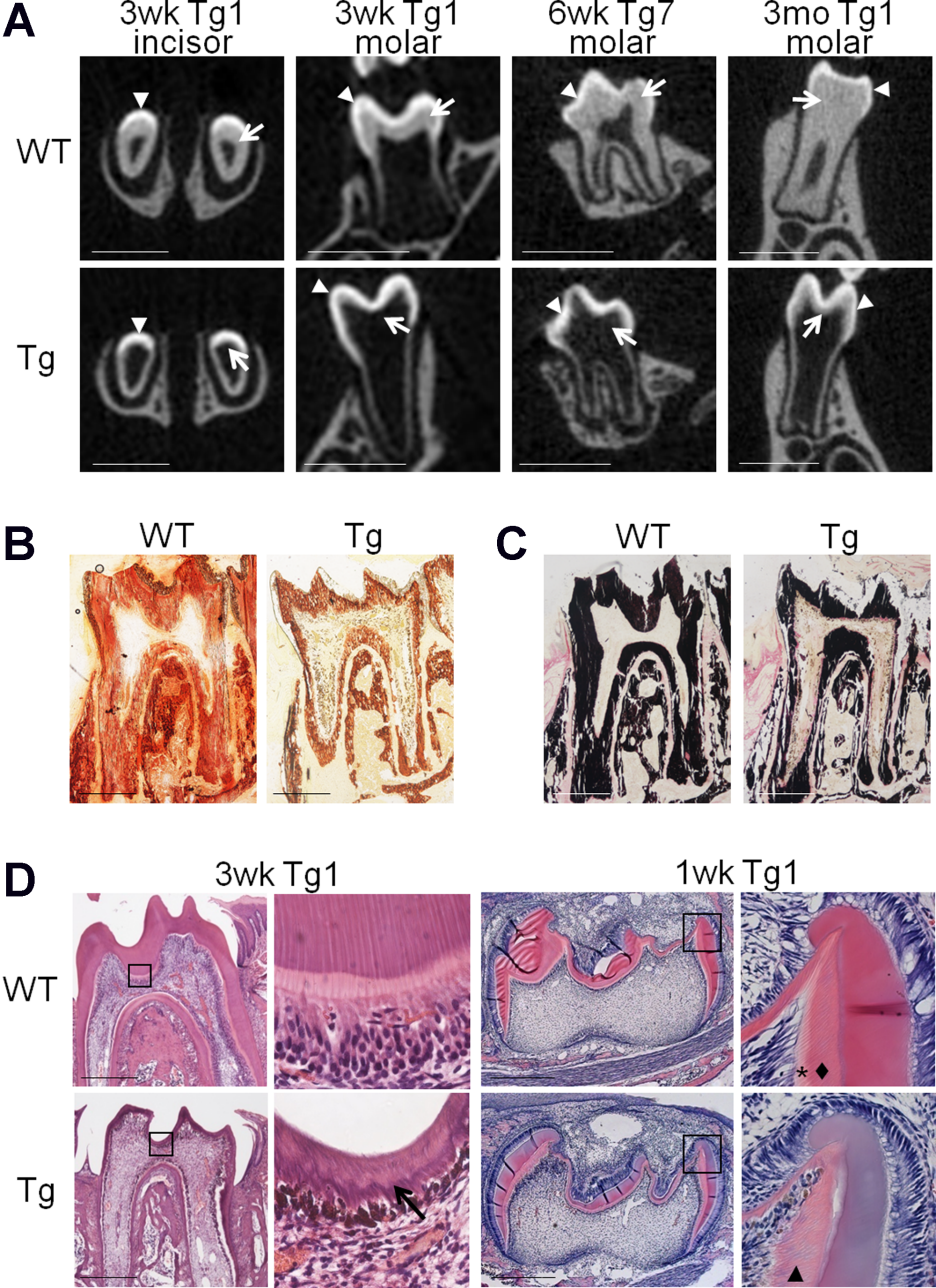
Defective dentin formation in *Col1a1-Trps1* mice. (*A*) µCT images (cross sections) of WT and transgenic teeth (Tg1 and Tg7 line) demonstrating a diminished dentin layer in transgenic mice. Arrowheads: enamel, arrows: dentin. Transgenic animals were fed with soft food to assure proper nutrition. Scale bars = 1 mm. (*B*) Alizarin red staining of undecalcified molar sections (3 weeks old) demonstrating reduced dentin layer in transgenic teeth. Scale bars = 500 µm. (*C*) Von Kossa staining of undecalcified molar sections (3 weeks old) demonstrating reduced dentin layer in transgenic teeth. Scale bars = 500 µm. (*D*) Histological analyses of molars showing abnormal dentin formation in transgenic mice. In the dentin of 3-week-old (3wk) transgenic animals (erupted teeth) an irregular mineralization front is observed (arrow). In the 1-week-old (1wk) WT animals (unerupted teeth) two distinctive layers of predentin and dentin are present (asterisk and diamond, respectively), while in transgenic animals only one layer is present (arrowhead). Black boxes indicate areas magnified on the images to the right. Scale bars = 500 µm.

The most common cause of the postweaning growth retardation and lethality is problems in feeding. Considering that our transgene is expressed in teeth and bone, we performed gross analyses of the craniofacial skeleton using µCT to assess potential problems with mineralization. µCT analyses of 3-week-old mice revealed that all *Col1a1-Trps1* mice presented with fractured incisors at the time of weaning (Fig. 1*G*). Additionally, cross-sectional images demonstrated dramatically reduced layers of dentin in both incisors and molars of transgenic versus age-matched WT littermates (Fig. 2*A*), which resulted in the enlarged dental pulp cavity. These results suggested that the growth retardation phenotype of *Col1a1-Trps1* mice might be caused by difficulties with mastication. To compensate for this, we provided *Col1a1-Trps1* mice with a soft food diet starting upon weaning, and continued throughout the life of the animals. Indeed, by reducing masticatory stresses on dentition, we were able to restore the normal lifespan of *Col1a1-Trps1* mice. In addition, there was no significant difference in the total body weight of WT and transgenic animals fed with soft food (Fig. 1*E*; Supplemental Fig. S2). These results indicate that severe malnutrition due to tooth fragility was the underlying cause of postweaning growth retardation and mortality seen in *Col1a1-Trps1* mice.

Although the soft food diet rescued postweaning growth retardation and lethality in *Col1a1-Trps1* mice, the notably abnormal dentin phenotype did not improve, indicating that dentin formation was not correctable over time in animals expressing *Trps1* in secretory odontoblasts. This was confirmed by µCT analyses of teeth from 6-week-old and 3-month-old *Col1a1-Trps1* mice fed with soft food showing a significantly reduced dentin layer in transgenic animals compared to WT littermates (Fig. 2*A*). The defective dentin phenotype was further confirmed by histological analyses of molars. Alizarin Red and von Kossa staining of undecalcified teeth demonstrated a reduced layer of mineralized dentin in *Col1a1-Trps1* mice compared to WT littermates (Fig. 2*B*, *C*). Notably less dentin in transgenic mice was also demonstrated on decalcified tissue sections (Fig. 2*D*). Additionally, at higher magnification of histological images the irregular mineralization front is clearly evident in the teeth of transgenic animals, suggesting that expression of *Trps1* in mature odontoblasts affects not only matrix secretion, but also matrix mineralization. Disturbed dentin mineralization can be readily seen in unerupted teeth. In WT P7 molars, distinctive layers of predentin and dentin are apparent, whereas in the teeth of transgenic animals, this organized predentin-dentin layered structure is diminished (Fig. 2*D*). In summary, overexpression of *Trps1* in secretory odontoblasts disturbs dentin formation, which leads to dramatically reduced dentin layers and tooth fragility. Concomitantly, µCT analyses of bone showed no significant differences between WT and transgenic mice (Supplemental Fig. S3). Thus, the phenotype of *Col1a1-Trps1* mice appears to recapitulate features of dentinogenesis imperfecta in humans, though the mechanism was not yet clear.

### Decreased levels of major noncollagenous dentin matrix proteins in *Col1a1-Trps1* mice

**Fig. 3:**
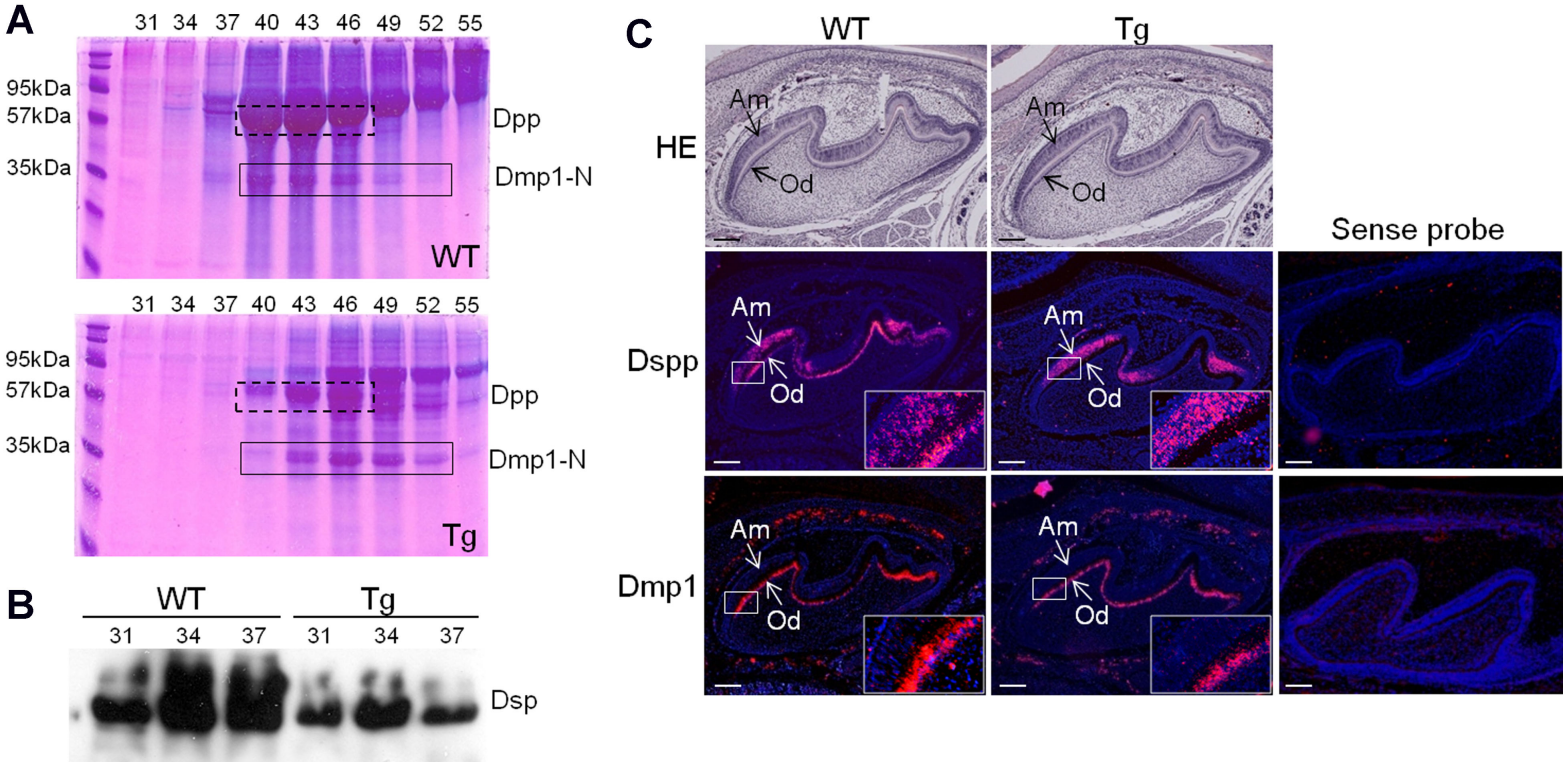
Decreased levels of DSP and DPP proteins, and *Dspp* gene expression in odontoblast of *Col1a1-Trps1* mice. (*A*) Detection of SIBLING proteins isolated from dentin of 8-week-old WT and transgenic mice. Proteins were extracted from dentin and separated into 120 fractions using ion-exchange chromatography. Selected fractions containing SIBLING proteins were analyzed on an SDS-PAGE gel. SIBLING proteins were visualized by Stains-All. Numbers above the gel image indicate the chromatographic protein fractions analyzed on the gel; these fractions contained DPP and DMP1 fragments. The dashed-line box marks DPP protein, the solid-line box marks the N-terminal fragment of DMP1 protein. In transgenic mice a total level of DPP is decreased in comparison with WT mice, whereas levels of DMP1 show no difference. (*B*) Western blot with DSP antibodies shows decreased levels of DSP in transgenic dentin. (*C*) RNA in situ hybridization with probes specific for *Dspp* and *Dmp1* (antisense molars, P2; sense molars P4). The boxed areas are magnified on the images in the right low corner of each respective image. In WT animals *Dspp* is expressed in both odontoblasts and ameloblasts, while in *Col1a1-Trps1* mice *Dspp* expression in odontoblasts is significantly reduced. There is no difference in expression of *Dmp1* between WT and transgenic odontoblasts. Od = odontoblasts; Am = ameloblasts. Scale bars = 100 µm.

In contrast to DGI-I, in which both bone and dentin are affected, presentation of DGI-II and DGI-III is restricted to dental abnormalities. These forms of DGI are caused by mutations in the *DSPP* gene. The *DSPP* gene encodes a protein that is proteolytically processed to form DSP and DPP, which are the most abundant noncollagenous proteins in the dentin matrix. To understand the molecular basis of abnormal dentin formation in *Col1a1-Trps1* mice, we compared the relative abundance of DSP, DPP, and other SIBLING proteins in the dentin matrices between WT and transgenic animals.^30^–^32^ These analyses showed dramatically reduced levels of DPP in transgenic animals (Fig. 3*A*). At the same time, levels of the N-terminal fragment of DMP1 showed no apparent differences as evaluated by Stains-All staining. Since DSP is less sensitive to Stains-All staining, we compared levels of DSP between WT and transgenic mice by Western blot. These analyses also showed dramatically decreased levels of DSP (Fig. 3*B*).

**Fig. 4:**
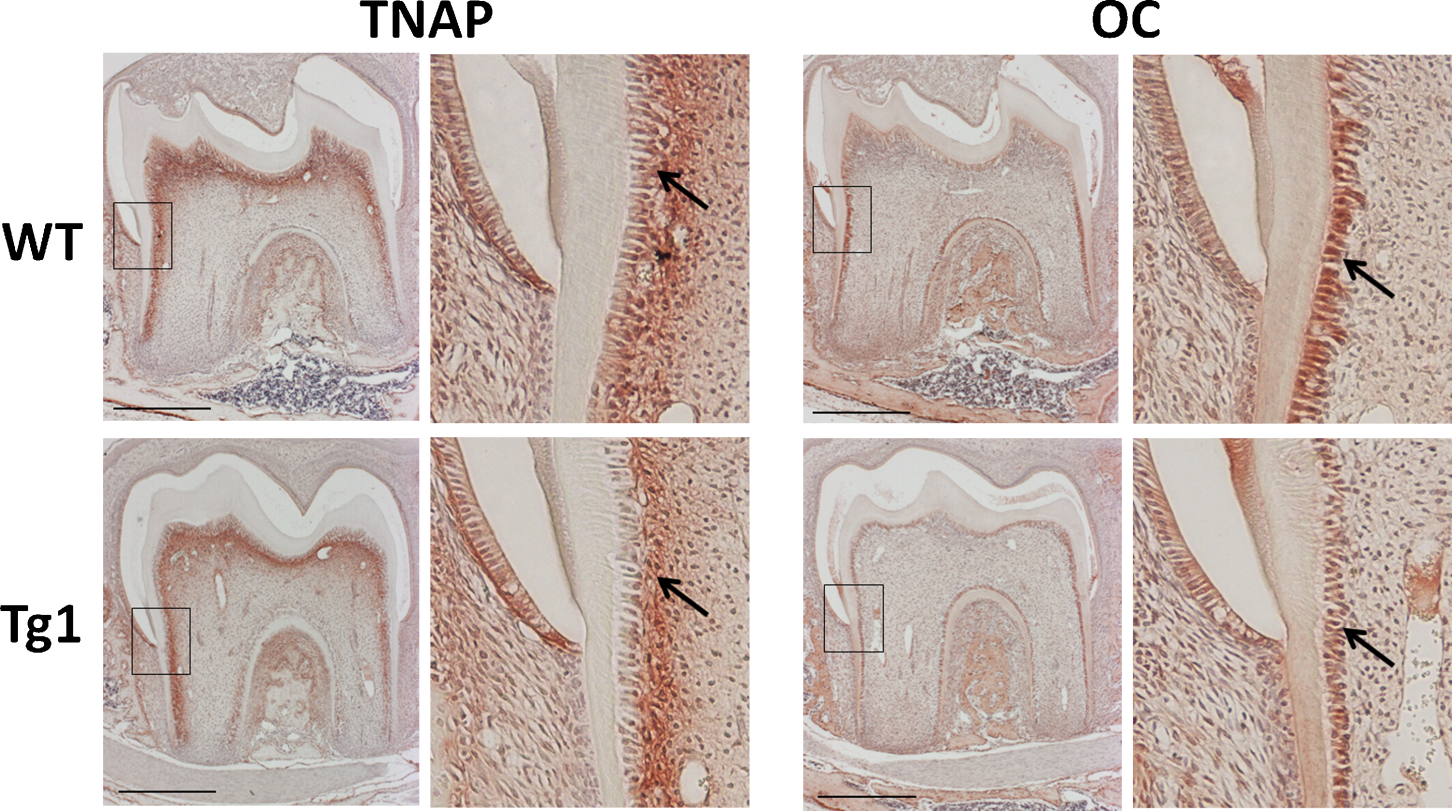
Immunohistochemical analysis of odontoblast markers in WT and *Col1a1-Trps1* molars (P14). Tissue nonspecific alkaline phosphatase (TNAP) and osteocalcin (OC) are detected both in WT and *Col1a1-Trps1* (Tg1) odontoblasts (arrows). Black boxes indicate area magnified on the images to the right of each respective image. Scale bars = 500 µm.

To evaluate whether sustained high expression of *Trps1* in secretory odontoblasts affects their differentiation status, we compared expression of other odontoblast markers in WT and transgenic teeth.^36^ Tissue nonspecific alkaline phosphatase (TNAP), which is expressed in preodontoblasts as well as in mature odontoblast in WT mice, was not changed in *Col1a1-Trps1* teeth. More importantly, osteocalcin (OC), which is expressed in late odontoblasts, showed no apparent differences in expression between WT and transgenic teeth either (Fig. 4). In summary, analyses of dentin matrix proteins demonstrated that overexpression of *Trps1* in mature odontoblasts leads specifically to decreased levels of the major noncollagenous dentin matrix proteins, DSP and DPP, without affecting the maturation of odontoblasts, because osteocalcin and DMP1 (markers of differentiated odontoblasts) remain unchanged.

### Trps1 represses *Dspp* expression through direct interaction with its promoter

Because both DSP and DPP proteins are expressed from the same gene, we next compared *Dspp* expression between WT and *Col1a1-Trps1* transgenic teeth. We did this by RNA ISH on unerupted molars from P2 mice. As expected, in WT mice the *Dspp* transcripts were detected in odontoblasts and also in ameloblasts, where *Dspp* is transiently expressed (Fig. 3*C*). In contrast, *Dspp* expression in odontoblasts of transgenic mice was dramatically reduced but it was unchanged in ameloblasts. We also compared expression of the *Dmp1* gene. The RNA ISH results were consistent with the protein analyses and no differences were observed in the expression of *Dmp1* between WT and transgenic odontoblasts (Fig. 3*C*). These results demonstrate that overexpression of *Trps1* in odontoblasts specifically represses expression of the *Dspp* gene in a cell autonomous fashion, suggesting that the *Dspp* gene might be a direct target of the Trps1 transcription factor.

**Fig. 5:**
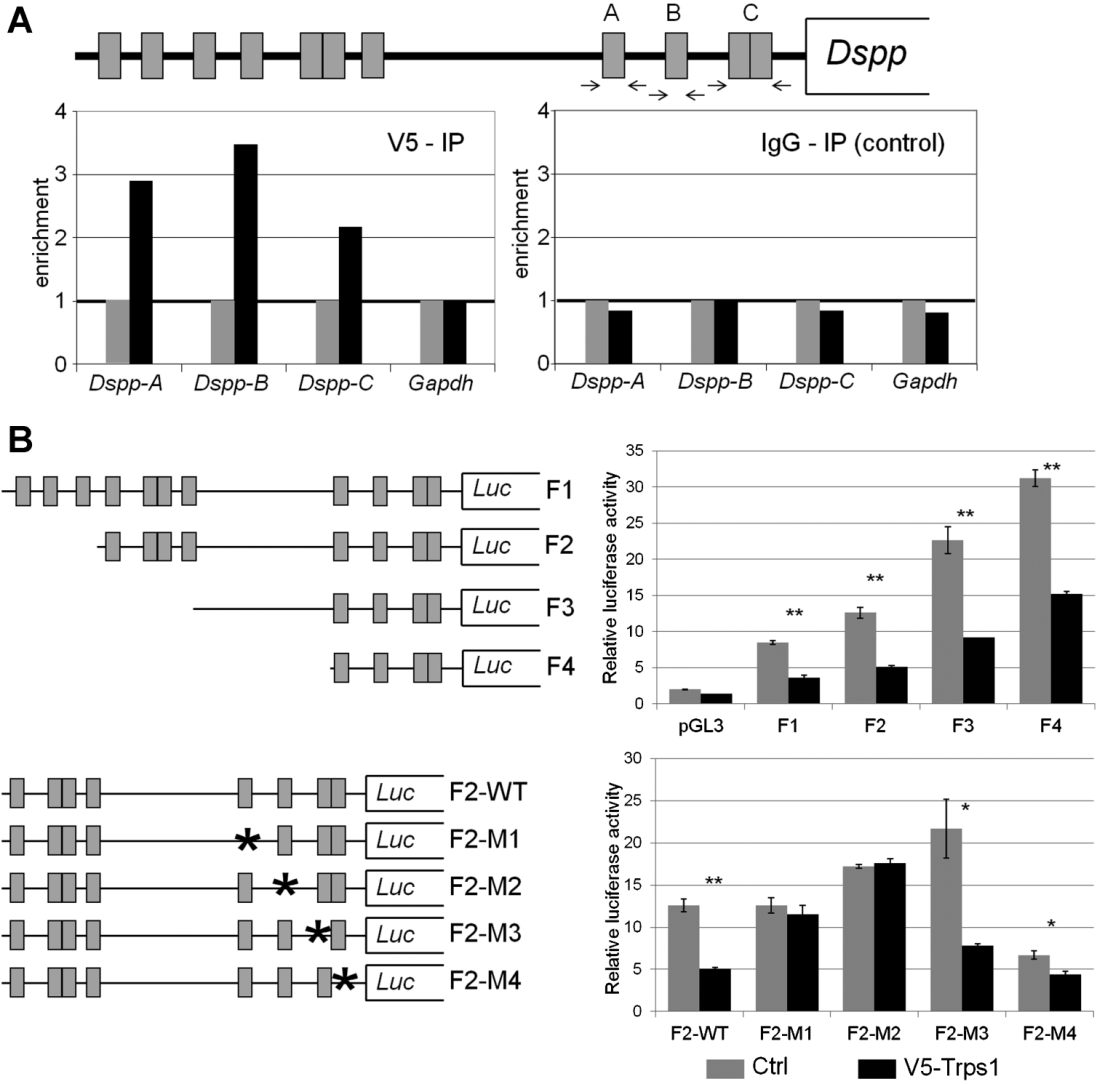
Role of putative GATA consensus elements in Trps1-mediated repression of the *Dspp*. (*A*) Interaction of Trps1 with putative GATA consensus sites in the mouse *Dspp* promoter analyzed by ChIP assay. Top: Schematic representation of the location of GATA elements (gray boxes) in the 1.5-kb fragment of the *Dspp* promoter. Arrows indicate localization of qPCR primers. Bottom: Results of qPCR on DNA immunoprecipitated with V5 antibodies (left panel) and control IgG antibodies (right panel) from the odontoblastic cell line stably overexpressing V5-tagged Trps1 (black bars) versus control cell line (gray bars). The values for the control cell line were arbitrarily set as 1 to better demonstrate the enrichment of the *Dspp* promoter sequences in the DNA immunoprecipitated from the cell line expressing V5-tagged Trps1. (*B*) Comparison of the *Dspp* promoter-luciferase reporter activity in control MDPC-23 cell line (Crtl, gray bars) and MDPC-23 cell line overexpressing V5-tagged Trps1 (V5-Trps1, black bars). Top: Schematics of *Dspp*-luciferase reporter constructs with different fragments of the *Dspp* promoter and their relative activities in MDPC-23 cell lines. Bottom: Schematic of the F2 *Dspp*-luciferase reporter constructs carrying single point mutations (asterisks) disrupting putative GATA consensus elements and their relative activity. Error bars represent the standard deviations of three independent experiments. Statistical significance was calculated using paired *t* test, *p ≤ 0.05, **p ≤ 0.01.

To test whether Trps1 can repress *Dspp* by directly regulating its promoter, we first screened the mouse *Dspp* promoter for consensus elements of GATA transcription factors. We performed computational analyses of the 1.5-kb fragment of the *Dspp* promoter and 5′ untranslated region (5′UTR) using the TFSEARCH program, ver. 3.1.^37^ These analyses identified 11 consensus binding sites for GATA transcription factors in the *Dspp* promoter (Fig. 5*A*). Although GATA binding sites are fairly common among gene promoters, their relatively high abundance in the *Dspp* promoter suggests that GATA transcription factors play an important role in regulation of *Dspp* expression. To test whether Trps1 can directly bind to four GATA consensus elements that are the most proximal to the transcriptional start site, we performed ChIP assays in the MDPC-23 mouse odontoblastic cell line^38^ that stably expresses V5-tagged Trps1. We immunoprecipitated DNA-protein complexes using V5 antibodies, and performed quantitative PCR to evaluate the relative occupancy of Trps1 on the selected GATA elements in the *Dspp* promoter. Three independent ChIP experiments demonstrated an average enrichment of 2-fold to 3.5-fold for the analyzed *Dspp* promoter fragments in the DNA immunoprecipitated from cells expressing V5-Trps1 as compared with the control cell line lacking exogenous Trps1 (Fig. 5*A*). There was no difference in the abundance of DNA fragments between these cell lines for the *Gapdh* reference gene. ChIP assay with control antibodies (normal mouse IgG) showed no differences, providing additional support for specific binding of Trps1 to the *Dspp* promoter (Fig. 5*A*).

To address the role of these GATA consensus elements in Trps1-mediated repression of the *Dspp*, we took an in vitro approach. First, we generated four reporter constructs (plasmids F1, F2, F3, and F4), in which firefly luciferase was expressed from various fragments of the mouse *Dspp* promoter (Fig. 5*B*). Control and V5-Trps1 expressing MDPC-23 stable cell lines were transfected with each of these constructs and a renilla luciferase-expressing plasmid to normalize for the transfection efficiency. Analyses of the reporter activity detected a 2-fold reduction in the activity of each reporter in MDPC-23 cells overexpressing *Trps1* in comparison with control cells. Furthermore, using the F2 *Dspp* reporter, we generated four additional reporter constructs carrying single point mutations in the putative GATA consensus elements that we analyzed for the Trps1 binding by ChIP (Fig. 5). Comparison of the mutant F2 *Dspp* reporters activity in control and *Trps1* overexpressing MDPC-23 cells demonstrated that eliminating the potential Trps1 binding site in either fragment A (construct F2-M1) or B (construct F2-M2) results in an increase in the reporter activity in V5-Trps1 MDPC-23 cells to levels comparable with control cells (Fig. 5*B*). This suggests that these putative GATA consensus binding sites are required for Trps1-mediated repression of the F2 *Dspp* reporter.

Collectively, these results demonstrate that Trps1 can directly bind GATA consensus sites in the *Dspp* promoter and this activity results in the repression of *Dspp* expression in vivo.

## Discussion

In our previous studies focused on the role of the Trps1 transcription factor in endochondral bone development, we uncovered that mice mutated for the *Trps1* gene present with an advanced mineralization of perichondrium phenotype.^25^ This suggests that *Trps1* is a negative regulator of mineralization. To understand the function of *Trps1* in mineralizing tissues we generated *Col1a1-Trps1* transgenic mice overexpressing *Trps1* specifically in osteoblasts and odontoblasts. Analyses of these mice demonstrated that Trps1 acts as a tissue-specific repressor of mineralization, with a strong effect on dentin, but with little influence on bone. Further, we determined that this context-dependent activity is specified by direct repression of the *Dspp* gene by the Trps1 transcription factor. As a result, the dentin matrix in *Col1a1-Trps1* mice exhibits dramatically reduced levels of DSP and DPP proteins, and, consequently, severely impaired mineralization. Hence, the phenotype of *Col1a1-Trps1* mice recapitulates main features of *Dspp* knockout (KO) mice and the human genetic disorder DGI.

Interestingly, the bone tissue of *Col1a1-Trps1* mice is not significantly affected, implying the tissue-specific effect of *Trps1* on mineralization. This specificity might be determined by different pools of target genes in osteoblasts and odontoblasts. Alternatively, Trps1 may repress genes critical for dentinogenesis but not for bone formation. Although dentin is similar to bone in terms of the extent of its mineralization and the expression of many of the same transcription factors and matrix proteins, high expression of the *Dspp* gene characterizes dentin as a unique mesenchyme-derived mineralized tissue.^39^–^41^ More importantly, the key role of *Dspp* in dentin formation is underscored by severe dentin impairment in cases where the *DSPP* gene is disrupted. For comparison, mutations in type I procollagen genes, *COL1A1* or *COL1A2*, affect both bone and dentin, demonstrating the requirement of collagen type I in both tissues. Thus, the downregulation of *Dspp* maybe be the major factor determining the tissue-specific *Trps1*-mediated repression of mineralization. Another important difference between bone and dentin is that dentin does not undergo remodeling, thus the quantity of dentin solely reflects odontoblast activity, whereas overall bone mass is a net result of the opposing activities of osteoblasts and osteoclasts. However, in our studies we did not measure the dynamic parameters of bone remodeling; therefore, we cannot exclude that the osteoblast activity is affected by *Trps1* overexpression.

Recently, Piscopo and colleagues^42^ showed that Trps1 binds elements in the osteocalcin promoter and represses osteocalcin promoter-driven reporter expression in various osteogenic cell lines. In addition, they demonstrated that osteocalcin expression is inversely correlated with levels of *Trps1* in bone marrow stromal cells undergoing osteogenic differentiation. Considering these data, and the fact that osteocalcin is also a marker of mature odontoblasts, we compared expression of osteocalcin in WT and *Col1a1-Trps1* teeth using immunohistochemistry. However, we did not observe significant differences between WT and transgenic mice, indicating that overexpression of *Trps1* does not affect osteocalcin expression in vivo. Additionally, these data, together with unchanged DMP1 expression, suggest that overexpression of *Trps1* does not cause the arrest of odontoblast differentiation; rather the dental phenotype of *Col1a1-Trps1* mice results from impaired odontoblast function.

Consistent with significantly reduced levels of DSPP, dentin abnormalities observed in *Col1a1-Trps1* mice are similar to those reported for *Dspp* KO mice, a mouse model of DGI. *Dspp* deficiency caused either by genetic disruption of the gene or by Trps1-mediated repression of *Dspp* expression results in enlarged pulp chambers and reduced dentin layers that are evident on µCT images of teeth. Moreover, at the histological level, decreased dentin and an irregular mineralization front are apparent in both mouse models, which further confirms defective dentin formation. In contrast to *Dspp* KO mice, we have not observed calcospherites or sporadic unmineralized areas in dentin of *Col1a1-Trps1* mice. This might be due to residual *Dspp* expression in transgenic mice, because, unlike *Dspp* KO mice in which expression of the gene is completely abolished, *Col1a1-Trps1* transgenic mice express very low levels of DSPP that accumulates in dentin over time. Alternatively, it is possible that *Trps1* overexpression in secretory odontoblasts may also affect expression of other genes, resulting in the phenotype of *Col1a1-Trps1* transgenic mice differing from the phenotype of *Dspp* KO mice.

DGI can be phenocopied in genetically manipulated mice by either mutating the *Dspp* gene or by diminishing *Dspp* expression indirectly by dysregulating other genes. The latter animal models provide valuable insight into the molecular mechanisms controlling the expression of this highly tissue-specific gene. In spite of its critical role in dentin formation, little is known about factors that determine spatial and temporal expression of *Dspp* in vivo. Animal studies have previously demonstrated that activation of TGF-β signaling represses the *Dspp* gene. Although TGF-β signaling is necessary for odontoblast differentiation,^17^, ^43^ overexpression of TGF-β1 in secretory odontoblasts results in decreased *Dspp* expression and the DGI phenotype in transgenic mice.^13^ Additional studies in odontoblastic cells have revealed that Smad3 mediates this inhibitory effect of TGF-β on the *Dspp* gene^16^; however, transcription factors that directly repress *Dspp* in response to TGF-β are unknown. Whether or not Trps1 is the mediator of TGF-β signaling in odontoblasts, remains to be determined. Trps1 is indeed a good candidate, given its high expression in dental mesenchyme and preodontoblasts in comparison with mature odontoblasts, in which it is expressed at low levels. This suggests that, akin to TGF-β signaling, Trps1 is involved in odontoblast differentiation, whereas in mature odontoblasts it suppresses dentin formation. In addition, the unusually high number of GATA elements in the *Dspp* promoter suggests an important role for this family of transcription factors in its regulation. Dramatically reduced levels of *Dspp* expression in odontoblasts overexpressing *Trps1* demonstrate that this GATA-type transcription factor is dominant over the activity of other transcription factors stimulating *Dspp* expression. These transcription factors remain to be determined, because, in spite of extensive in vitro analyses of various transcription factors as candidate regulators of *Dspp* expression, none has been validated in vivo.^15^, ^41^, ^44^–^46^

Our transgenic mouse approach, in which high *Trps1* expression is sustained in secretory odontoblasts, may help to understand the molecular pathology underlying dentin defects caused by transcriptional dysregulation. Such a mechanism has been identified in Ambras syndrome, a rare disorder caused by a translocation with a break point in the *Trps1* promoter, leading to the aberrant pattern of *Trps1* expression.^47^ Unlike TRPS patients that are characterized by sparse slow-growing hair, the most prominent feature of Ambras syndrome is extensive growth of hair over the entire body (hypertrichosis). Interestingly, although not described in detail, Ambras patients also present with a dental phenotype, indicating that dysregulation of the spatial and/or temporal pattern of *Trps1* expression results in dental abnormalities in humans. Whether the aberrant expression pattern of *Trps1* contributes to other disorders with dental presentations remains to be determined. For example, the genetic cause of spondylometaphyseal dysplasia with DGI (odontochondrodysplasia [ODCD]) is still unknown.^48^ Interestingly, in this disorder both endochondral bone formation and dentinogenesis are affected, pointing to involvement of a gene that functions in both tissues. In our studies, we identified the Trps1 transcription factor, expressed during endochondral bone as well as tooth development, as a potent inhibitor of dentin formation, thus it is plausible that Trps1 and a gene responsible for ODCD function in the same pathway. Our results not only identified the *Dspp* gene as the first Trps1 target gene in odontoblasts, but also shed light on the in vivo regulation of this gene, which is critical for dentin formation. Thus, revealing the Trps1 molecular network in odontoblasts will certainly contribute to greater understanding of the molecular mechanisms of dentinogenesis and associated human dental disorders.
