# Supplementary figures and images for: Transcriptional Repression of the Dspp Gene Leads to Dentinogenesis Imperfecta Phenotype in Col1a1-Trps1 Transgenic Mice
Source: J Bone Miner Res. 2012 Apr 16;27(8):1735–45. doi: 10.1002/jbmr.1636 (PMC3399940; doi:10.1002/jbmr.1636)

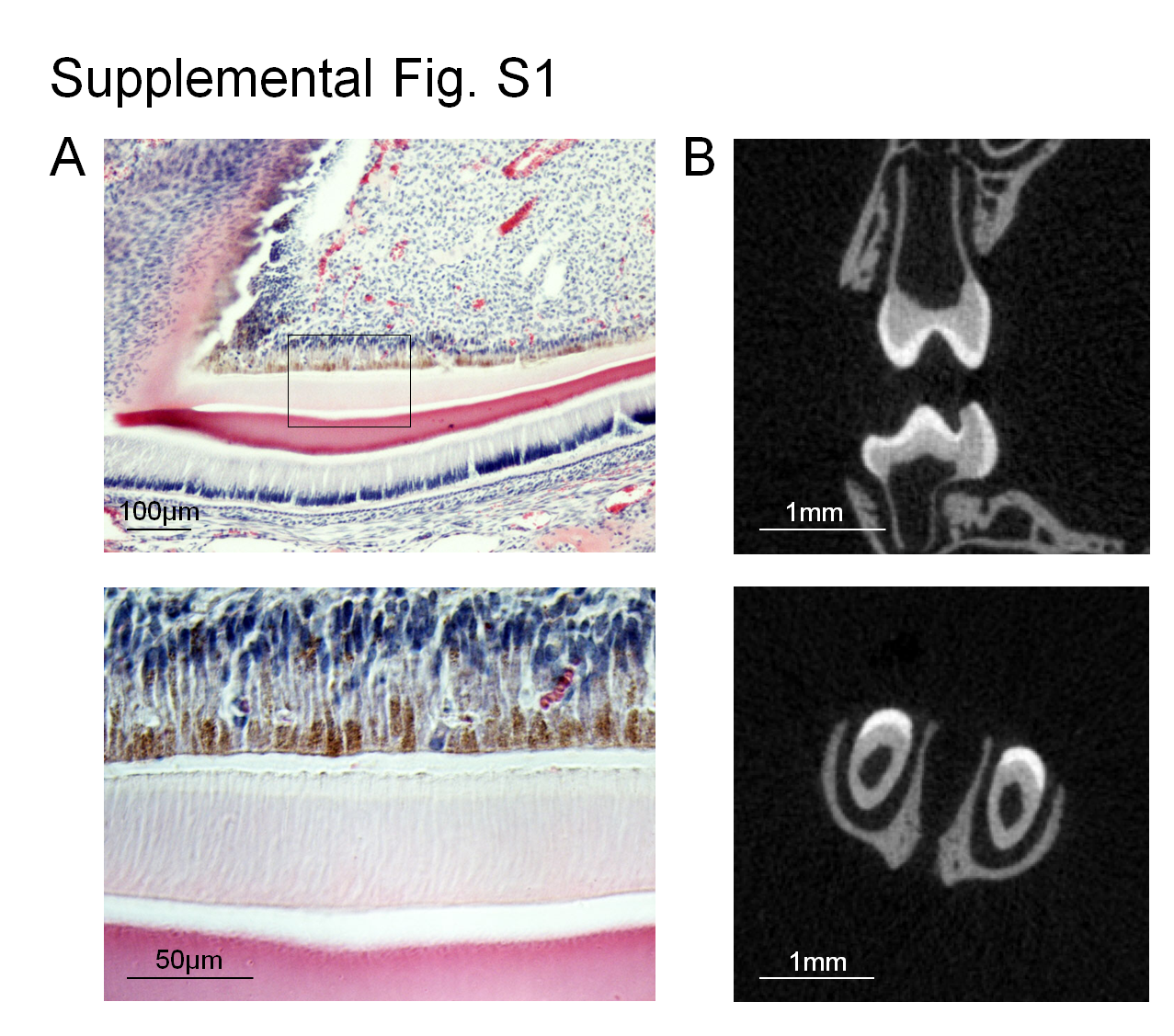

Supplement: Supplementary file 1 [file jbmr0027-1735-SD1.tif]

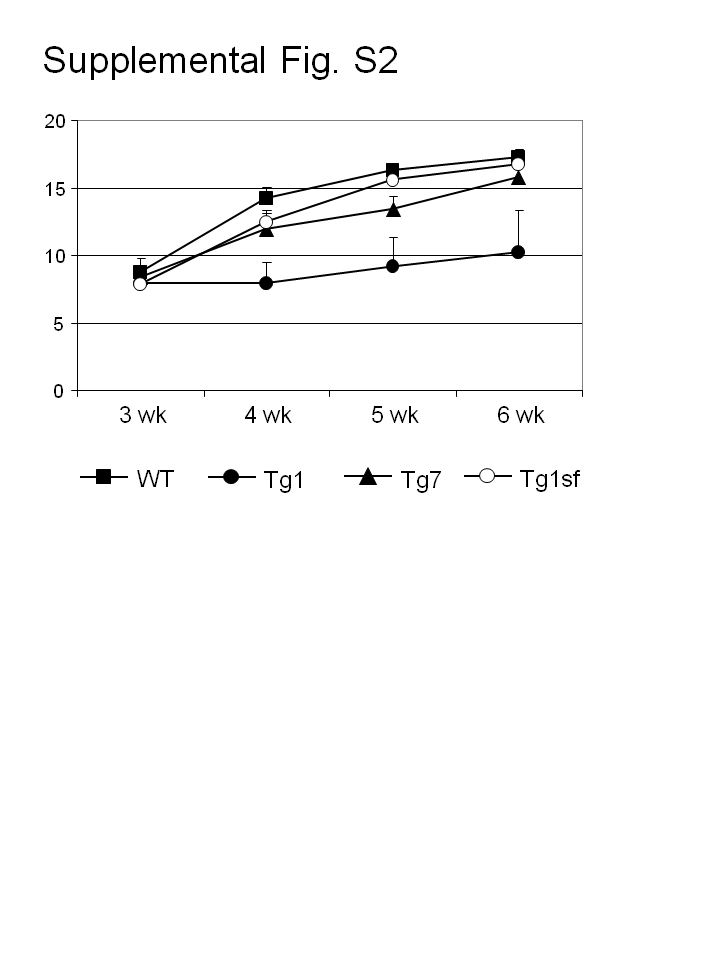

Supplement: Supplementary file 2 [file jbmr0027-1735-SD2.tif]

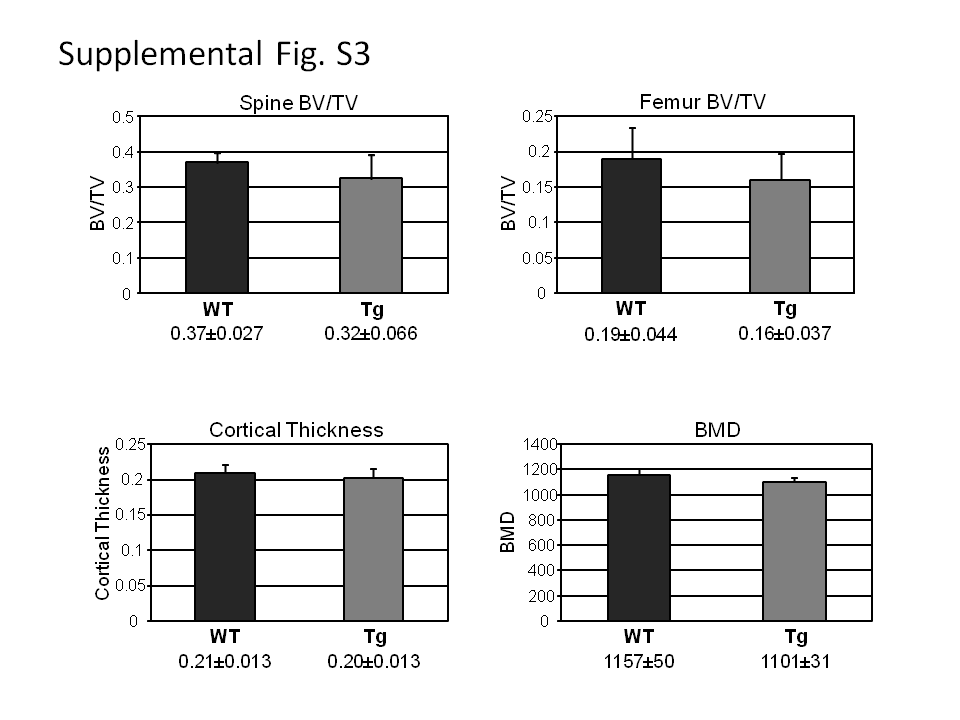

Supplement: Supplementary file 3 [file jbmr0027-1735-SD3.tif]
