# Supplementary material for: Transcriptional Repression of the Dspp Gene Leads to Dentinogenesis Imperfecta Phenotype in Col1a1-Trps1 Transgenic Mice
Source: J Bone Miner Res. 2012 Apr 16;27(8):1735–45. doi: 10.1002/jbmr.1636 (PMC3399940; doi:10.1002/jbmr.1636)
Supplement: Supplementary file 4 [file jbmr0027-1735-SD4.doc]

**Legends to Supplementary Figures**

**Supplementary Figure S1.** Expression of the tyrosinase minigene in odontoblasts of transgenic mice does not affect dentin formation (3wk old mice generated for a project unrelated to presented work).

**A.** H&E staining of incisor demonstrates pigment deposition in odontoblasts. Black boxes indicate areas magnified on the image below. There is no apparent aberration of dentin formation.

**B.** Micro-CT analyzes show no apparent adverse effects on dentin mineralization in molars (top) and incisors (bottom).

**Supplementary Figure S2.** Post-weaning growth retardation of *Col1a1-Trps1* mice due to malnutrition.

Weight curves of WT and *Col1a1-Trps1* mice (females, N=10) demonstrate growth retardation of transgenic animals. The phenotype is more severe in the Tg1 transgenic line that overexpresses *Trps1* at higher levels than the Tg7 line. The growth retardation is corrected by providing a soft food diet (open circles: Tg1 females fed with soft food, N=4).

**Supplementary Figure S3.** Micro-CT analyses of lumbar spine vertebrae (L4) and femur of 3mo males.

Although minor decrease in bone volume over tissue volume was detected in *Col1a1-Trps1* mice (N=6) in comparison with WT (N=6), the difference is not statistically significant. Data are expressed as mean values +/- standard deviation. Statistical significance was calculated using Student’s *t*-test.
